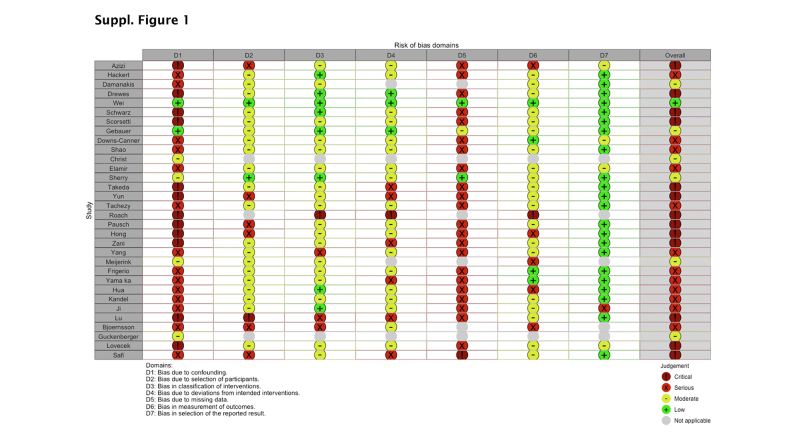# Defining oligometastatic pancreatic cancer: a systematic review and critical synthesis of consensus

**DOI:** 10.1016/j.esmoop.2023.102067

**Published:** 2023-11-20

**Authors:** C.-S. Leonhardt, T. Stamm, T. Hank, G. Prager, O. Strobel

**Affiliations:** 1 Department of General Surgery, Division of Visceral Surgery, Medical University of Vienna, Vienna; 2 Institute of Outcomes Research, Center for Medical Data Science, Medical University of Vienna; 3 Ludwig Boltzmann Institute for Arthritis and Rehabilitation, Vienna; 4 Department of Medicine I, Division of Oncology, Medical University of Vienna, Vienna, Austria

**Keywords:** pancreatic cancer, oligometastasis, oligometastatic disease, consensus definition, local consolidative treatment

## Abstract

**Background:**

Small retrospective series suggest that local consolidative treatment (LCT) may improve survival in oligometastatic pancreatic ductal adenocarcinoma (PDAC). However, no uniform definition of oligometastatic disease (OMD) in PDAC exists; this impedes meaningful conclusions.

**Patients and methods:**

A systematic literature search using PubMed, Web of Science, and Cochrane CENTRAL registries for studies and protocols reporting on definitions and/or LCT of OMD in PDAC was performed. The primary endpoint was the definition of OMD. Levels of agreement were categorized as consensus (≥75% agreement between studies), fair agreement (50%-74%), and absent/poor agreement (<50%).

**Results:**

After screening of 5374 abstracts, the full text of 218 studies was assessed, of which 76 were included in the qualitative synthesis. The majority of studies were retrospective (*n* = 66, 87%), two were prospective studies and eight were study protocols. Studies investigated mostly liver (*n* = 38, 51%) and lung metastases (*n* = 15, 20%). Across studies, less than one-half (*n* = 32, 42%) reported a definition of OMD, while 44 (58%) did not. Involvement was limited to a single organ (consensus). Additional criteria for defining OMD were the number of lesions (consensus), metastatic site (poor agreement), metastatic size (poor agreement), treatment possibilities (poor agreement), and biomarker response (poor agreement). Liver OMD could involve three or fewer lesions (consensus) and synchronous disease (fair agreement), while lung metastases could involve two or fewer lesions and metachronous disease (consensus). The large majority of studies were at a high risk of bias or did not include any control groups.

**Conclusion:**

Definitions of OMD were not used or varied widely between studies hampering across-study comparability and highlighting an unmet need for a consensus. The present study is part of a multistep process that aims to develop an interdisciplinary consensus on OMD in pancreatic cancer.

## Highlights

• An increasing number of studies are reporting on the treatment of oligometastatic pancreatic cancer.
• This systematic review reveals that definitions of oligometastatic pancreatic cancer vary widely.
• Oligometastasis was mostly characterized by <3 synchronous lesions in the liver and two or fewer metachronous lesions in the lungs.
• Different definitions hinder cross-study comparability and underscore the unmet need for a consensus.

## Background

Pancreatic ductal adenocarcinoma (PDAC) is a deadly malignancy with rising incidence.^1^ Surgery offers the only potential chance of cure; however, only 10%-20% of patients are initial candidates for surgical resection while the majority of patients presents with locally advanced disease or distant metastasis.

In recent years, multimodal chemotherapy regimens, as well as advanced surgical techniques, significantly increased the chance of potentially curative surgery, resulting in resection of previously unresectable locally advanced tumors in up to 60% of cases.^2^ In parallel, perioperative mortality rates decreased.^3^ However, the large majority of cancer-related deaths in PDAC continues to be driven by metastatic spread.^4^

In PDAC, distant metastases are widely regarded as a contraindication to local consolidative treatment (LCT) with curative intention including surgery, radiofrequency ablation, and stereotactic body radiotherapy.^5^, ^6^, ^7^ Macroscopically visible systemic tumor spread seems to render a localized treatment approach futile, making systemic chemotherapy the treatment of choice.^5^ However, LCT is increasingly investigated in patients with PDAC presenting with a limited number of metastases based on the concept of oligometastatic disease (OMD).^8^

OMD, initially proposed by Hellmann and Weichselbaum in 1995, is understood as an intermediate state between localized and polymetastatic disease in which limited metastatic spread has occurred, yet the tumor lacks the ability for widespread systemic dissemination.^9^, ^10^, ^11^ Some clinical evidence supports the concept of an oligometastatic state in PDAC: on the one hand, survival seems to differ in metastatic pancreatic cancer dependent on the site of initial metastasis,^12^^,^^13^ on the other hand, resection of isolated metachronous pulmonary metastases has been associated with improved survival compared with other sites of metastases in retrospective series.^14^^,^^15^ Yet, no widely accepted definition of oligometastatic PDAC exists and variable definitions lead to controversy and confusion.^16^^,^^17^

Importantly, standardized definitions are a prerequisite for future treatment progress. The primary aim of this systematic review was to identify definitions of OMD in PDAC and synthesize a consensus based on these. The secondary aim encompassed the provision of a comprehensive descriptive synopsis of survival.

## Methods

This study was registered in the PROSPERO registry of systematic reviews (CRD42023439102) and conducted according to the Synthesis Without Meta-analysis (SWiM) reporting guidelines.^18^

### Eligibility criteria

All studies reporting on OMD in PDAC were eligible for inclusion, including randomized trials, nonrandomized trials, study protocols, and case series with six patients or more. No age or period restrictions were applied. Studies that did not report a definition of OMD and/or did not report on LCT of OMD were excluded. Non-English language studies, narrative reviews, meta-analyses, conference abstracts, and case series with less than six patients were also excluded. Furthermore, studies solely focusing on extra-regional lymph node metastases were excluded. Registry studies were included in the qualitative synthesis if they provided a definition of OMD. However, they were excluded from any investigations concerning the effect on survival after LCT.

### Search strategy

A systematic search using PubMed, Web of Science, and Cochrane CENTRAL registries was carried out and our last update was on 17 October 2022. No limitations on the time frame were set. The search strategy for MEDLINE is depicted in the Supplementary Materials, available at https://doi.org/10.1016/j.esmoop.2023.102067; the other search strategies are available upon request.

The search strategy was validated by cross-comparison of the results with a recent systematic review on LCT in oligometastatic PDAC and manual checking if selected references were included in the retrieved results.^19^

### Screening and data extraction

Covidence systematic review software (Veritas Health Innovation, Melbourne, Australia) was used to manage references. Titles and abstracts of all references were screened independently by two reviewers (CSL and TH). In case of disagreement, a discussion was initiated among the investigators until a consensus was reached (defined as ≥75% agreement). Additional studies were identified by manually checking the reference lists of selected studies.

For data extraction, a specific extraction form was designed and pilot-tested using selected studies. Extracted basic data included first author, publication year, country, sample size, study type, study period, age, and sex.

In addition, definitions of OMD, data on the treatment of the primary tumor and metastases, existence of a control group, median overall survival (OS), median disease-free interval (DFI), size of metastases, number of metastatic sites, number of affected organ(s), time pattern of metastases, performance status, biochemical response, imaging modality, and imaging response were retrieved.

### Critical appraisal

The Risk Of Bias In Non-randomised Studies - of Interventions (ROBINS-I) tool was used to judge the quality of the eligible studies.^20^ This tool was selected as it is specifically designed for observational studies and most eligible studies were anticipated to be of nonrandomized design. The domains included bias due to confounding, bias due to selection of participants, bias in classification of interventions, bias due to deviations from intended interventions, bias due to missing data, bias in measurement of outcomes, and bias in selection of the reported result. Each potential source of bias was graded as low, moderate, serious, and critical.

The risk of bias in the respective domains and overall bias was assessed by two reviewers (CSL and TH) working independently. Disagreements were resolved through consultations among the investigators as described previously.

### Data synthesis

Based on the data extraction of the first half of the eligible articles, it was determined that a meta-analysis based on pooled hazard ratios of survival outcomes was not appropriate because of substantial methodological and statistical heterogeneity. All data synthesis without meta-analysis was performed according to the Cochrane Handbook and the SWiM guidelines, respectively.^18^^,^^21^

Consensus was defined as specified in a recent systematic review and meta-analysis and analogous to previous studies in oligometastatic esophagogastric cancer as well as oligometastatic non-small-cell lung cancer (NSCLC).^22^, ^23^, ^24^, ^25^ In brief, ≥75% agreement between studies was considered ‘consensus’, agreement of 50%-74% was considered ‘fair agreement’, and agreement <50% was considered ‘absent/poor agreement’.^22^

For the definition of consensus, studies were grouped based on the metastatic site. As only a small number of appropriate studies was expected per group, a minimum of two studies was required for a definition of consensus. Subsequently, the following four categories were identified for metastatic sites: (i) liver metastases, (ii) lung metastases, (iii) liver and/or lung metastases, and (iv) other metastatic sites.

To identify the criteria most commonly used for defining OMD, frequency counts were used, resulting in five different categories:

(i) Treatment possibilities
(ii) Size of metastatic lesion
(iii) Affected organ
(iv) Number of metastases
(v) CA 19-9 biomarker response in cases of systemic therapy before LCT

Subsequently, a consensus was synthesized as described above.

### Statistics

As most studies did not include a control group or did not provide measures of effect between intervention and control groups, no formal meta-analysis could be conducted.

For survival analysis, the median OS after resection was used. OS times between diagnosis and the resection of primary tumor were equated for the calculation of the weighted median of medians. The weighted median of medians was calculated according to the method described by McGrath and colleagues.^26^

All statistical analysis was conducted using R (R Foundation, Vienna, Austria). A two-sided *P*-value <0.05 was considered significant.

### Patient and public involvement

Patients as well as representatives of patient organizations were involved during the process of this systematic review. Their ideas and opinions regarding potentially relevant differences in metastatic PDAC were taken into account in personal discussions during meetings. Furthermore, suggestions concerning the avoidance of technical terms were considered whenever possible without compromising the scientific content.

## Results

**Figure 1:**
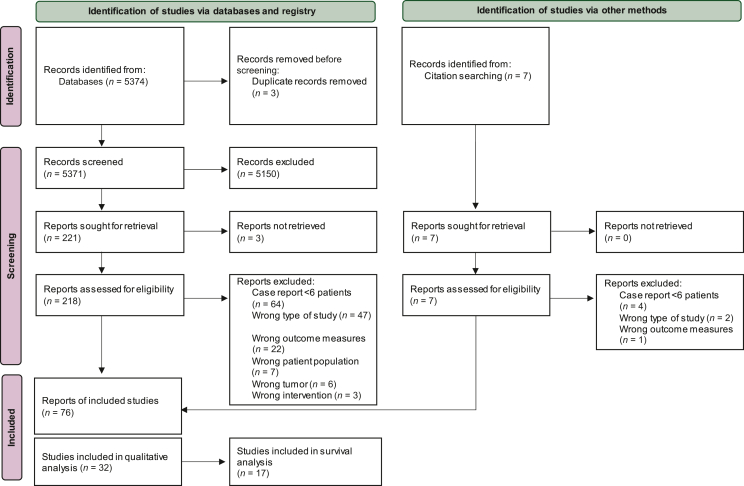
Study flowchart.

After screening of 5374 abstracts, 218 full-text articles were assessed for eligibility (Figure 1). Finally, 76 studies were identified, which provided a definition of OMD in PDAC and/or reported LCT of OMD (Table 1). Most studies were retrospective (*n* = 66, 87%),^2^^,^^7^, ^8^, ^9^, ^10^, ^11^, ^12^, ^13^, ^14^, ^15^, ^16^, ^17^, ^18^, ^19^, ^20^, ^21^, ^22^, ^23^, ^24^, ^25^, ^26^, ^27^, ^28^, ^29^, ^30^, ^31^, ^32^, ^33^, ^34^, ^35^, ^36^, ^37^, ^38^, ^39^, ^40^, ^41^, ^42^, ^43^, ^44^, ^45^, ^46^, ^47^, ^48^, ^49^, ^50^, ^51^, ^52^, ^53^, ^54^, ^55^, ^56^, ^57^, ^58^, ^59^, ^60^, ^61^, ^62^, ^63^, ^64^, ^65^, ^66^, ^67^, ^68^, ^69^, ^70^, ^71^, ^72^, ^73^, ^74^, ^75^, ^76^, ^77^, ^78^, ^79^ while two were prospective^80^^,^^81^ and eight were study protocols.^82^, ^83^, ^84^, ^85^, ^86^, ^87^, ^88^, ^89^ After the exclusion of study protocols and registry studies, the studies comprised a total of 2365 patients and most reported single-center data.

**Table 1:** Study characteristics

Variable		Total (*n* = 76), *n* (%)	Definition OMD (*n* = 32), *n* (%)
**Study type**			
	Prospective	2 (3)	1 (3)
	Retrospective	66 (87)	24 (75)
	Study protocol	8 (11)	7 (22)
**Number of patients**^a^			
	Locoregional treatment	2365	1351
**Time point**			
	Synchronous	30 (39)	15 (47)
	Metachronous	21 (28)	3 (9)
	Synchronous and metachronous	16 (21)	8 (25)
	Not specified/NA	9 (12)	6 (19)
**Number of organs**			
	1	69 (91)	30 (94)
	>1	1 (1)	1 (3)
	Not specified/NA	6 (8)	1 (3)
**Induction chemotherapy (synchronous metastasis)**			
	Yes	11 (24)	5 (22)
	No	35 (76)	18 (78)

NA, not applicable; OMD, oligometastatic disease.

a Excluding study protocols and registry studies.

The majority (*n* = 69, 91%) of studies involved one metastatic site, while one study involved metastasis in multiple organs^82^ and six studies did not report the number of organs involved.^41^^,^^45^^,^^50^^,^^51^^,^^53^^,^^84^ Thirty (40%) studies encompassed synchronous metastasis,^29^^,^^31^^,^^33^^,^^34^^,^^40^^,^^42^, ^43^, ^44^^,^^46^, ^47^, ^48^, ^49^^,^^51^^,^^52^^,^^56^^,^^60^^,^^61^^,^^64^^,^^69^^,^^70^^,^^72^^,^^73^^,^^75^^,^^80^^,^^83^^,^^88^, ^89^, ^90^, ^91^, ^92^ whereas 21 (28%) involved metachronous metastases^13^^,^^27^^,^^35^^,^^37^^,^^53^^,^^57^, ^58^, ^59^^,^^63^^,^^65^^,^^67^^,^^71^^,^^74^^,^^76^^,^^77^^,^^79^^,^^93^, ^94^, ^95^, ^96^ and 16 (21%) studies involved both synchronous and metachronous metastases.^2^^,^^14^^,^^30^^,^^32^^,^^36^^,^^38^^,^^39^^,^^54^^,^^62^^,^^66^^,^^68^^,^^78^^,^^81^^,^^85^^,^^86^^,^^97^ The remaining nine studies either did not specify the time point of OMD or no specification was applicable due to the study design.^16^^,^^17^^,^^28^^,^^41^^,^^45^^,^^50^^,^^82^^,^^84^^,^^87^ In total, 11 (15%) studies involved some kind of preoperative treatment,^33^^,^^44^^,^^46^^,^^49^^,^^51^^,^^61^^,^^73^^,^^83^^,^^85^^,^^89^^,^^91^ while 35 (46%) did not and the remaining ones did not specify it or it was not applicable due to the study design.^2^^,^^14^^,^^29^, ^30^, ^31^, ^32^^,^^34^^,^^36^^,^^38^, ^39^, ^40^^,^^42^^,^^43^^,^^47^^,^^48^^,^^52^^,^^54^^,^^56^^,^^60^^,^^62^^,^^64^^,^^66^^,^^68^, ^69^, ^70^^,^^72^^,^^75^^,^^78^^,^^80^^,^^81^^,^^86^^,^^88^^,^^90^^,^^92^^,^^97^

### Definitions of oligometastatic disease

Among the 76 studies included in the analysis, only 32 (42%) provided a definition of OMD,^2^^,^^16^^,^^17^^,^^28^^,^^32^^,^^36^^,^^39^^,^^46^, ^47^, ^48^, ^49^^,^^54^, ^55^, ^56^^,^^68^^,^^72^^,^^77^^,^^82^, ^83^, ^84^^,^^86^, ^87^, ^88^, ^89^^,^^91^^,^^96^ whereas the majority (*n* = 44, 58%) failed to do so^13^^,^^14^^,^^27^^,^^29^, ^30^, ^31^^,^^33^, ^34^, ^35^^,^^37^^,^^38^^,^^41^, ^42^, ^43^, ^44^, ^45^^,^^50^, ^51^, ^52^, ^53^^,^^57^, ^58^, ^59^, ^60^^,^^62^^,^^63^^,^^65^, ^66^, ^67^^,^^70^^,^^71^^,^^74^^,^^76^^,^^78^, ^79^, ^80^^,^^86^^,^^90^, ^91^, ^92^, ^93^, ^94^, ^95^^,^^97^ (Table 1). Of note, three (9%) studies reported a definition of OMD based on survival outcomes or imaging data without context to a therapeutic intervention.^16^^,^^17^^,^^32^ The collective sample size across all the studies amounted to 1351 patients. Among these studies, the majority focused on synchronous metastasis (*n* = 15, 46.8%).^16^^,^^46^, ^47^, ^48^, ^49^^,^^56^^,^^64^^,^^69^^,^^72^^,^^73^^,^^75^^,^^83^^,^^88^^,^^89^^,^^91^ Three (9%) examined metachronous metastasis,^55^^,^^77^^,^^96^ and eight (25%) studies examined synchronous and metachronous metastasis.^2^^,^^32^^,^^36^^,^^39^^,^^54^^,^^68^^,^^81^^,^^85^ Six (19%) studies did not specify the time point of metastasis.^16^^,^^17^^,^^28^^,^^82^^,^^84^^,^^87^. Preoperative treatment was delivered in seven (22%) studies before the administration of LCT with curative intent.^46^^,^^49^^,^^73^^,^^83^^,^^85^^,^^89^^,^^91^

**Figure 2:**
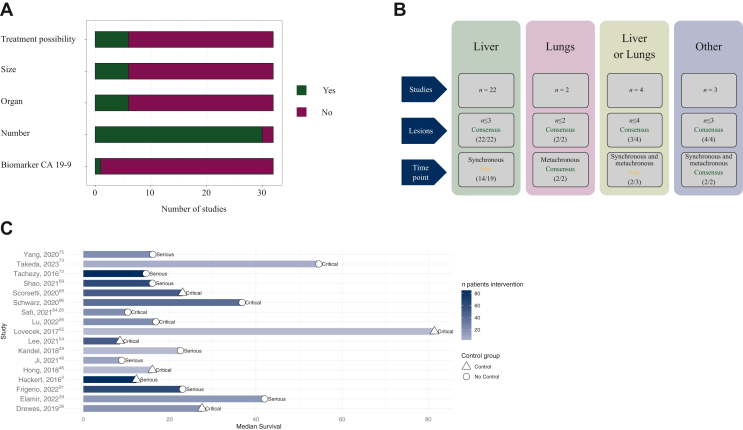
(A) Criteria used to define oligometastatic pancreatic cancer. (B) Graphical summary of consensus definitions of oligometastatic pancreatic cancer by metastatic site. (C) Harvest plot depicting median postresection survival after resection of the primary tumor. Blue shading indicates the sample size, icons indicate whether or not the respective study contains a control group. Labels ‘serious’, ‘critical’ next to icons indicate the overall risk of bias based on the Risk Of Bias In Non-randomised Studies - of Interventions (ROBINS-I) tool.

All studies reported OMD affecting a single organ, except the study of Guckenberger and colleagues, which included a total of 1-5 metastases not necessarily restricted to the same organ.^82^ One study did not specify the number of organs and some studies combined liver and lung metastases in their reports.^84^ Criteria other than the organ affected in definitions of OMD were treatment strategies (e.g. amenable to resection), size of the metastatic lesion, number of metastatic lesions, as well as CA 19-9 biomarker response after treatment (Figure 2A). Among the studies reporting definitions of OMD, imaging modalities used to diagnose OMD were mainly computed tomography (57%) and/or magnetic resonance imaging (50%) and/or [18F]2-fluoro-2-deoxy-D-glucose-positron emission tomography/computed tomography based (27%), but were often not specified (33%). Eight studies were prospective trials and study protocols, including the basket trial of Sherry and colleagues^81^ (Supplementary Table S1, available at https://doi.org/10.1016/j.esmoop.2023.102067).^81^, ^82^, ^83^, ^84^, ^85^^,^^87^^,^^88^^,^^98^

### Consensus for liver oligometastasis

In total, 22 studies reported on oligometastatic liver disease (Figure 2B, Table 2). Studies were mainly retrospective (*n* = 16),^2^^,^^17^^,^^28^^,^^36^^,^^46^, ^47^, ^48^^,^^54^^,^^56^^,^^64^^,^^69^^,^^72^^,^^73^^,^^75^^,^^91^^,^^96^ five studies were study protocols,^83^^,^^85^^,^^87^, ^88^, ^89^ and one was a Surveillance, Epidemiology, and End Results (SEER) database study.^61^ The retrospective studies comprised a total of 692 patients. Equal or less than three liver metastases were considered OMD in 22 of 22 studies, thus reaching a consensus (100% agreement). In total, 19 studies described the time pattern of metastases, of which 14 considered liver OMD as synchronous metastases (fair agreement, 74%).^46^, ^47^, ^48^^,^^56^^,^^61^^,^^64^^,^^69^^,^^72^^,^^73^^,^^75^^,^^83^^,^^88^^,^^89^^,^^91^ For the three studies that reported DFI before the diagnosis of metachronous metastasis, the weighted median DFI was 17.0 months [95% confidence interval (CI) 17.0-18.4 months].^2^^,^^54^^,^^96^ No consensus based on the number of affected hepatic lobes could be synthesized.

**Table 2:** Study characteristics of pancreatic cancer with liver metastases, lung metastases, and multiple/other sites of OMD

First author	Year	Country	Study type	Study period	Treatment primary tumor	Treatment metastases	Median OS (months)	Patients (locoregional treatment)	Definition OMD	Metastatic sites	Time point
Lee^54^	2021	United States	Retrospective	2008-2018	Resection	Radiotherapy	8.5 (after radiotherapy)	52	Yes	Liver	Synchronous and metachronous
Ji^48^	2021	China	Retrospective	2010-2019	SBRT	SBRT	8.9	34	Yes	Liver	Synchronous
Yamanaka^17^	2021	Japan	Retrospective	2001-2019	NA	NA	9.8 (H1 classification)	39	Yes	Liver	NA
Pausch^61^	2021	Germany	Registry study (SEER)	2010-2015	NA	Resection	10	259	Yes	Liver	Synchronous
Safi^64^	2021	Germany	Retrospective	2006-2019	Resection	Resection	10.3	17	Yes	Liver	Synchronous
Hua^47^	2017	China	Retrospective	2012-2015	Not specified	RFA	11.4	102	Yes	Liver	Synchronous
Hackert^2^	2017	Germany	Retrospective	2001-2014	Resection	Resection	12.3 (from metastasectomy)	85	Yes	Liver	Synchronous and metachronous
Tachezy^72^	2016	Germany	Retrospective	1994-2014	Resection	Resection	14.5	69	Yes	Liver	Synchronous
Azizi^28^	2011	Germany	Retrospective	2002-2007	No resection	TACE	16	32	Yes	Liver	NA
Hong^46^	2018	United States	Retrospective	2010-2016	Resection	Resection	16	7	Yes	Liver	Synchronous
Shao^69^	2021	China	Retrospective	2009-2018	Resection	Resection	16	50	Yes	Liver	Synchronous
Yang^75^	2020	China	Retrospective	2012-2017	Resection	Resection	16.1	23	Yes	Liver	Synchronous
Lu^56^	2022	China	Retrospective	2017-2020	Resection	RFA	16.8	15	Yes	Liver	Synchronous
Frigerio^91^	2022	Italy	Retrospective	2008-2020	Resection	NA	23	52	Yes	Liver	Synchronous
Drewes^36^	2019	Germany	Retrospective	2010-2017	Resection	High-dose brachytherapy	27.5	16	Yes	Liver	Synchronous and metachronous
Schwarz^96^	2020	Multinational	Retrospective	2004-2015	Resection	Resection	36.8	25	Yes	Liver	Metachronous
Takeda^73^	2022	Japan	Retrospective	2013-2020	Resection	Resection	54.6	10	Yes	Liver	Synchronous
Zanini^78^	2018	United States	Study protocol	2018-2022	Resection	Resection/microwave ablation	NA	15	Yes	Liver	Synchronous
Wei^89^	2019	China	Study protocol	2018-2023	Resection	Resection	NA	NA	Yes	Liver	Synchronous
Meijerink^87^	2020	Netherlands	Study protocol	2020-2023	No resection	NA	NA	18	Yes	Liver	NA
Gebauer^99^	2021	Germany	Study protocol	2021-2025	Resection	Resection	NA	150	Yes	Liver	Synchronous
Björnsson^85^	2022	Multinational	Study protocol	2022-2027	Resection	Resection/RFA/IRE	NA	64	Yes	Liver	Synchronous and metachronous
Lovecek^55^	2017	Czech Republic	Retrospective	2006-2013	Resection	Resection	81.4	3	Yes	Lung	Metachronous
Yun^77^	2022	Korea	Retrospective	2007-2018	Resection	Resection	6.2^a^	13	Yes	Lung	Metachronous
Kandel^49^	2018	United States	Retrospective	2005-2015	Resection	Resection/ablation	22.5	6	Yes	Liver and lung	Synchronous
Damanakis^16^	2019	Germany	Retrospective	2008-2018	None	None	19.4	10	Yes	Liver and lung	NA
Scorsetti^68^	2020	Italy	Retrospective	2013-2017	Resection	SBRT	23 (from the time of SBRT)	33	Yes	Liver, lung, and LN	Synchronous and metachronous
Christ^32^	2022	Switzerland	Retrospective	January to December 2020	None	None	Not specified	637	Yes	Bone, liver, lung, brain, and LN	Synchronous and metachronous
Elamir^39^	2022	United States	Retrospective	2011-2020	Resection	SBRT	42	20	Yes	Liver, lung	Synchronous and metachronous

H1 classification, Japanese Classification of Colorectal, Appendiceal, and Anal Carcinoma 3^rd^ English Edition; IRE, irreversible electroporation; LN, extra-regional lymph nodes; NA, not applicable; OMD, oligometastatic disease; OS, overall survival; RFA, radiofrequency ablation; SBRT, stereotactic body radiotherapy; SEER, Surveillance, Epidemiology, and End Results Program; TACE, transarterial chemoembolization.

a 5-year survival rate.

### Consensus for lung oligometastasis

Only two studies defined lung oligometastases^55^^,^^77^ (Table 2). Lung OMD was considered as two or fewer metastatic lesions (consensus, 100%) and metachronous time pattern (consensus, 100%; Figure 2B). The weighted median DFI was 35.4 months (95% CI 18-35.4 months) until diagnosis of metachronous metastasis.

### Consensus for other locations of oligometastasis

The trial by Sherry and colleagues^81^ was excluded from this analysis as it only contained one patient with pancreatic cancer. Subsequently, three studies were identified, which included multiple sites of metastasis or did not specify a specific anatomical location^32^^,^^82^^,^^84^ (Table 2). In these, three or fewer metastatic lesions (consensus, 100%) were considered OMD while two of these studies specified the time pattern of metastases. Here, synchronous and/or metachronous (consensus, 100%) were considered OMD.^32^^,^^81^ In addition, four studies were identified, which involved liver and lung metastases without specifically differentiating between the two^16^^,^^39^^,^^49^^,^^68^ (Table 2). In these, four or fewer metastatic lesions (consensus, 75%) and synchronous and/or metachronous time patterns (fair agreement, 67%) were considered OMD.

### Critical appraisal and survival analysis

All studies were at a high risk of bias based on the ROBINS-I tool (Supplementary Figure S1, available at https://doi.org/10.1016/j.esmoop.2023.102067). The weighted median OS of all the 17 studies that reported a definition of OMD and resection of the primary tumor was 16 months (95% CI 12.3-23.0 months).^2^^,^^36^^,^^39^^,^^46^^,^^48^^,^^49^^,^^54^, ^55^, ^56^^,^^64^^,^^68^^,^^69^^,^^72^^,^^73^^,^^75^^,^^91^^,^^96^ Nine of these studies reported survival data for a control group, resulting in a weighted median OS of 7.5 months (95% CI 7.5-10.4 months).^2^^,^^39^^,^^48^^,^^56^^,^^69^^,^^72^^,^^73^^,^^75^^,^^96^ Notably, the other studies did not include a control group or reported its survival. A harvest plot including studies defining OMD and reporting median OS is presented in Figure 2C.

## Discussion

An increasing number of studies investigate oligometastatic PDAC. However, no uniform definition exists. Based on the present systematic review oligometastatic PDAC encompasses either three or fewer synchronous liver metastases or two or fewer metachronous lung metastases. Importantly, of the 76 studies identified, only 32 provided a definition of OMD. Noteworthy, none of the identified eight study protocols registered at ClinicalTrials.gov used the same definition of oligometastatic PDAC.

In several cancers, LCT of metastases limited in number and location results in a survival benefit in comparison to systemic therapy only, for example, colorectal cancer and NSCLC.^100^, ^101^, ^102^ In colorectal liver metastases, positive lymph node status, extrahepatic disease, short DFI, high preoperative tumor markers, size of the primary tumor, and others have been identified as negative predictors of poor survival.^103^ Moreover, stereotactic body radiotherapy resulted in a survival benefit in different types of oligometastatic cancers in a multicenter randomized controlled trial.^104^

Novel multimodal preoperative therapy in pancreatic cancer have led to an increased number of case reports and retrospective series that in highly selected pancreatic cancer patients’ LCT of metastases might result in favorable outcomes as well.^44^^,^^105^, ^106^, ^107^, ^108^, ^109^, ^110^, ^111^, ^112^, ^113^, ^114^ In particular, complete response to chemotherapy based on imaging criteria has encouraged clinicians to seek resection of metastases in addition to the primary tumor as part of individualized treatment strategies.^44^^,^^115^^,^^116^

However, a lack of an accepted definition of OMD is a relevant hindrance to any meaningful conclusions. Other tumor entities lead the way. An international panel of experts recently proposed a definition of oligometastatic esophagogastric cancer (Table 3).^24^ OMD was defined as one or two metastases in either the liver, lung, retroperitoneal lymph nodes, adrenal glands, or soft tissue/bone. Interestingly, OMD additionally applied to no evidence of progressive disease at restaging after 18 weeks of systemic therapy.^24^ Similarly, definitions of OMD have been developed for NSCLC as well as breast cancer.^9^^,^^117^^,^^118^ Notably, the National Comprehensive Cancer Network (NCCN) guidelines and the eight edition of the tumor–node–metastasis (TNM) system already incorporate an oligometastatic stage into the classification of NSCLC.^119^

**Table 3:** Definitions of oligometastatic disease in other cancer entities

Included criteria	Breast cancer	Non-small-cell lung cancer	Esophagogastric cancer
Location	Not necessarily the same organ	≤3 organs	Liver, lung, retroperitoneal lymph nodes, adrenal gland, soft tissue, or bone
Number of metastases	≤5	≤5	1-2
Local therapy	Amenable to local treatment	Technically resectable	—
Synchronous/metachronous	—	Synchronous	Both
Others	Low-volume disease	Mediastinal lymph nodes not counted	Additionally, if there is no progression at restaging after a median of 18 weeks of systemic therapy

Two previous papers proposed a definition of OMD in PDAC. Damanakis et al.^16^ defined an oligometastatic state as CA 19-9 <1000 U/ml, four or fewer metastases in the liver/lung, and response or stable disease after first-line chemotherapy based on survival outcome without LCT. Applying these criteria, a subgroup of patients with significantly extended OS was identified. A recent study from Japan identified 54 patients who underwent staging laparoscopy or gastrointestinal bypass surgery as suffering from oligometastatic pancreatic cancer based on CA 19-9 <2000 U/ml and either one to four liver metastases or peritoneal metastasis localized to the adjacent peritoneum or limited to the distant peritoneum.^17^ Oligometastatic patients had a significantly longer survival compared with a polymetastatic control cohort.

For surgical treatment of metastases, two minimal prerequisites must be fulfilled: (1) both primary tumor and metastatic lesions must be technically resectable and (2) patients must be in good general health to be able to tolerate a large resection. Importantly, to justify LCT, the median OS should at least be >11 months which can be achieved with FOLFIRINOX-based systemic treatment in metastatic PDAC.^120^

In the absence of randomized trials, no high-quality evidence exists for any LCT strategy. The Chinese Study Group of Pancreatic Surgery (CSPAC) launched the multicenter phase III CSPAC-1 trial (NCT03398291) which aims to enroll 300 patients with pancreatic cancer with three or fewer synchronous liver metastases undergoing simultaneous resection after induction chemotherapy.^89^ The German HOLIPANC study is a phase II trial launched in 2021 and includes patients with five or fewer synchronous liver metastases treated with preoperative chemotherapy and then undergoing resection if the restaging shows stable disease or response according to RECIST 1.1 (NCT04617457).^99^ METAPANC is a phase III study investigating the effect of intensified chemotherapy followed by surgery for oligometastatic liver disease defined as three or fewer resectable liver metastases.^121^ Furthermore, laboratory values as well as Eastern Cooperative Oncology Group (ECOG) status were considered in the published protocol. However, no registration on ClinicalTrials.gov has been published so far, hence the study was not considered in the data synthesis of the present systematic review.

The present systematic review has several limitations, which need to be recognized. First, the total number of identified studies was small, far smaller than that of other tumor entities.^24^^,^^25^ This most likely reflects the current clinical practice as well as the unique tumor biology of PDAC. In addition, due to a high degree of methodological heterogeneity, no formal meta-analysis was conducted. Accordingly, we adhered to the SWiM guidelines for systematic reviews without meta-analysis, which have been developed to improve the reporting of findings and validity when using alternative synthesis methods.^18^ Furthermore, some of the identified studies did not provide sufficient data granularity for further analysis or did not clearly delineate between synchronous or metachronous metastases in the reported results.^16^^,^^39^^,^^49^^,^^68^

To correctly identify OMD, imaging plays a key role. The EORTC recently published recommendations on preferred imaging modalities of OMD in several cancers.^122^ By contrast, in the identified studies, only rarely the specific imaging modality to define OMD was reported.

Future work is necessary to identify the underlying molecular differences that determine the capacity of the tumor cell to metastasize. The concept of OMD implicitly assumes a hierarchical metastatic progression. Recent work indicates that the loss of the tumor suppressor gene *SMAD4* is associated with metastatic capacity and preclinical models support differences in metastatic capacity based on transcriptomic subtypes.^123^^,^^124^ As most patients who present with initially limited metastases quickly suffer from widespread systemic dissemination, it will be important to develop molecular biomarkers that will allow clinicians to tailor treatment to underlying tumor biology.^76^ Evidence from a xenograft mouse model indicates that the microRNA-200 family might be involved in the regulation between an oligometastatic and a polymetastatic state.^125^

### Conclusion

In summary, the majority of studies reporting on OMD do not provide a definition, leading to controversy and hampering comparability. We systematically reviewed the literature and synthesized a consensus definition of oligometastatic PDAC. It will be necessary to establish reliable criteria upon which patients will most likely benefit from LCT of metastases. A standardized definition of oligometastatic pancreatic cancer is an important step in that direction.

## Figures and Tables

**Supplementary Figure 1:**