# Supplementary material for: Defining oligometastatic pancreatic cancer: a systematic review and critical synthesis of consensus
Source: ESMO Open. 2023 Nov 20;8(6):102067. doi: 10.1016/j.esmoop.2023.102067 (PMC10774968; doi:10.1016/j.esmoop.2023.102067)
Supplement: Supplementary Table [file mmc1.docx]

**Suppl. Table 1:** Registered clinical trials with a definition of oligometastatic disease

| **Registration number** | **Investigator** | **Country /region** | **Status** | **OMD definition** | **Number of lesions** | **Organ** | **Systemic pretreatment** | **Treatment primary tumor** | **Mandated imaging** |
| --- | --- | --- | --- | --- | --- | --- | --- | --- | --- |
| **NCT03599765** | Tang | USA | Recruiting | Number | ≤5 | NA | NA | NA | NA |
| **NCT03398291** | Yu | China | Recruiting | Number | ≤3 | Liver | Yes | Resection | CT/MRI |
| **NCT02892305** | Zani | USA | Active, not recruiting | Number, organ | ≤3 | Liver | No | Resection | NA |
| **NCT02950025** | Roach | USA | Early termination | Number, treatment | ≤3 | NA | NA | NA | CT/MRI/PET-CT |
| **NCT04498767** | Guckenberger | Europe | Recruiting | Number, size, treatment | ≤5, ≤5cm | Not limited to single organ^1^ | NA | NA | NA |
| **NCT04612530** | Meijerink | Netherlands | Recruiting | Number, organ | NA | ≥1 liver^2^ | NA | NA | NA |
| **NCT04617457** | Gebauer | Germany | Recruiting | Number | ≤5 | Liver | Yes | Resection | CT/MRI |
| **NCT05271110** | Björnsson | Denmark/Sweden | Not yet recruiting | Number, size | <4, <5cm | Liver | Yes | Resection | NA |

**Legend:** ^1^suitable for MRI guided stereotactic body radiotherapy; ^2^ maximum number determined by multidisciplinary tumor board; NA: not applicable/ not specified, OMD: oligometastatic disease.
