# Supplementary material for: Defining oligometastatic pancreatic cancer: a systematic review and critical synthesis of consensus
Source: ESMO Open. 2023 Nov 20;8(6):102067. doi: 10.1016/j.esmoop.2023.102067 (PMC10774968; doi:10.1016/j.esmoop.2023.102067)
Supplement: Supplementary Figure 1 [file mmc2.docx]

**Suppl. File 1*:*** Search strategy for Medline.

*(“pancreatic cancer” OR “pancreatic adenocarcinoma” OR “pancreas adenocarcinoma” OR “pancreatic ductal adenocarcinoma”) AND (metastatic OR “liver metastases” OR “hepatic metastases” OR oligometastatic) AND (surgery OR resection OR ablation OR radiotherapy OR metastasectomy OR “local therapy” OR “local treatment”) “irreversible electroporation” OR “stereotactic body radiotherapy” OR “microwave ablation” OR “selective internal radiation therapy”)*
